# Impact of Land Use/Cover Change on Yangtze River Delta Urban Agglomeration Ecosystem Services Value: Temporal-Spatial Patterns and Cold/Hot Spots Ecosystem Services Value Change Brought by Urbanization

**DOI:** 10.3390/ijerph16010123

**Published:** 2019-01-04

**Authors:** Zhigang Li, Zishu Sun, Yangjie Tian, Jialong Zhong, Wunian Yang

**Affiliations:** 1 College of Management Science, Chengdu University of Technology, No.1 Dongsan Road, Erxian Bridge, Chenghua District, Chengdu 610059, China; lizhigang@mail.cdut.edu.cn; 2 Key Laboratory of GeoSpatial Information Technology of Ministry of Land and Resources, Chengdu University of Technology, No.1 Dongsan Road, Erxian Bridge, Chenghua District, Chengdu 610059, China; ywn@cdut.edu.cn; 3 College of Earth Sciences, Chengdu University of Technology, No.1 Dongsan Road, Erxian Bridge, Chenghua District, Chengdu 610059, China; TianYangJie_CDUT@163.com (Y.T.); jialogn@gmail.com (J.Z.)

**Keywords:** LUCC, urbanization, ecosystem services value, cold/hot spots analysis, spatial pattern, Yangtze River Delta Urban Agglomeration

## Abstract

Land use/cover change (LUCC) from increased urbanization significantly impacts regional ecosystem services. Based on a cold/hot spots analysis, this paper used grain yield, food prices, price index statistics, and a land use thematic map to study the impact of LUCC on four ecosystem services values (ESVs) in the Yangtze River Delta urban agglomeration, and determine the spatial differences resulting from the rapid urbanization LUCC. The correlation between the four ecosystem services was then studied and sensitivity analyses conducted to investigate whether any changes in the ESVs could lead to unacceptable unit value transfer uncertainties. It was found that most urban land was converted from farmland, and that before 2000, the total ESVs and the regulating services values (RSVs) increased significantly, after which it declined, the provisioning services values (PSVs) declined year on year, the habitat services value (HSV) and cultural and amenity services value (CSV) declined sharply after 2000, and the spatial distribution of the four ESVs were significantly different. Over time, it was found that the hot spots were shrinking and the cold spots were spreading. The provisioning services were found to be negatively correlated with habitat services and cultural and amenity services, the regulating services were weakly positively correlated with the provisioning services and significantly positively correlated with the habitat services and cultural and amenity services, and the habitat services were significantly positively correlated with cultural and amenity services. In the Yangtze River Delta urban agglomeration, the water area is the most important for the total ESVs, followed by non-bush forest. Paddy field is ranked third. Dryland, bush, grassland, and wetland are less important. The importance of barren land is almost zero. This research provides the government with a scientific basis from which to formulate spatial planning and environmental protection policies for ecological sustainable development in the Yangtze River Delta urban agglomeration.

## 1. Introduction

LUCC (Land use/cover change) programs involve the study of land surface changes, and were inspired by the NASA (National Aeronautics and Space Administration) Earth Science Program [1]. As land is the most essential ecosystem element, LUCC can have a significant impact on regional ESVs (ecosystem services values) [2,3,4,5]. The physical aspects of land (biological), such as the rocks, soil, vegetation, and water, are referred to as land cover, and the human uses, such as arrangements, activities, and inputs, are referred to as land use [6,7]. Land use changes inevitably affect the ecosystem, biodiversity, as well as communities [8]. Land cover can change because of natural phenomena, such as weathering, glaciers, and vegetation succession; natural disasters, such as earthquakes and mudslides; and human activities [9]. When these changes occur, the land can return to equilibrium; however, some land restoration efforts, such as recovering from desertification, can take a long time [10] and require significant resources. Land use/land cover changes can cause significant environmental impacts, such as soil degradation, deforestation, a loss of biodiversity, and declines in both water quantity and water quality [11], all of which affect the ability of the ecosystems to provide the natural products and services critical to human survival and well-being [12]. Consequently, the ESVs has become an important indicator to assess whether a region is able to attain sustainable development [13]. It has, therefore, become important to identify, quantify, and evaluate all ecosystem services changes [14], with quantitative assessments of land-use changes being the main approach [15,16]. Some scholars have been advocated for integrating ecosystem services into land use decisions [17,18,19], and it can help to promote interaction between researchers and policymakers to develop more effective policies to improve the ecological environment, but there are few methods and demonstrations in practice. So, Liang et al. have developed a method, integrating ESVs to balance future ecosystem-service benefit and risk to optimize investment in land for ecological conservation in land use planning [19]. 

The ESVs concept, which assesses the ecosystem-service benefits that humans receive from natural systems, was first proposed by Costanza et al. The ecosystem services economic value is determined from the interactions between the ecosystem supply and the human/social needs [15]. As natural systems are vitally important to economic well-being, the ecosystem value includes an assessment of the many ways human and natural systems interact, with the goal being to ensure that “nature” is fully reflected in government decision-making processes [20]. The benefit transfer assessment method uses existing valuation studies or data to estimate the ESVs at a site and then transfers these results to ESVs at similar locations [21]. In 1997, Constanza et al. took the lead and estimated the system functional value of the global biosphere ecosystem services. The publication of the results in the Nature magazine attracted widespread attention and prompted increased research interest in ecosystem services economic values assessments. Since that time, there has been significant research published on the impact of land use change on the ESVs on global scales [22], national scales, [23], and regional scales [24], with many watershed scale studies [25,26,27] as well as multi-scale comparative studies [28]. Research has also focused on assessing individual ESVs for forests [29,30,31], grasslands [32], farmland [33,34], wetlands [35,36], and marine environments [37]. Therefore, because of these extensive studies, there have also been many assessment methods developed, such as cost-benefit analysis [38], ecosystem services willingness to pay assessments [39], value transfers [21,40], public participation in GIS (PPGIS) methods [41], selection experiments [42], conditional valuations [43], and multiple or mixed methods (group reviews, semi-structured interviews, and analytical techniques, such as factor analysis) [44]. Li et al. estimated the impact of land-based land changes on the ESVs using the product of the area and a coefficient to reflect the land use impact [45]. Fei et al. revised the ESVs calculation method based on biophysical and socio-economic factors, and reflected the land use change impact on the ESVs in terms of space and quantity [46]. Wang et al. studied the driving factors behind land use change and the ESVs responses in Hengduan Mountains along the elevation gradient [47]. Cao et al. analyzed the ESVs gains and losses resulting from the rapid urbanization of the coastal areas of Zhejiang Province from a land use change perspective [48]. Arowolo et al. assessed the ESVs changes based on the land use/land cover dynamics in different administrative regions in Nigeria [49]. Yu et al. quantitatively evaluated the ESVs and the influence of human activities in the upper reaches of the Yangtze River, and proposed related planning methods [50]. Most previous research has quantitatively assessed land use change impacts on the ESVs. However, other studies have examined ESVs changes from the perspective of LUCC [48], have integrated ecosystem services into land use impact assessment methods to differentiate the different land use types [51], and have analyzed regional spatial differences [23]. However, few studies have considered the spatial heterogeneity of the LUCC impact on the various ESVs types and have offered a visual representation of these spatial changes. 

Rapid economic development and urban expansion has resulted in significant changes in land use/cover, which has in turn resulted in changes in the ecosystem structures around cities, reducing the ESVs [52]. In particular, the growth in the Yangtze River Delta urban agglomeration in China has been at the expense of farmland, with the urban ecological function significantly weakening and threatening the overall stability of the ecological environment [53]. In recent years, governments at all levels have sought to control the decline in the Yangtze River Delta urban agglomeration ecological environment. However, as no quantitative assessments have been conducted to assess the advantages and disadvantages of the ecological environmental changes or the accompanying regional changes due to the growing urban sprawl, the ecosystem services trends and spatial patterns remain unclear.

This paper takes the Yangtze River Delta urban agglomeration as a research area to explore the potential impact of the LUCC on four ESVs; provisioning services, regulating services, habitat services, and cultural and amenity services; and to assess their spatial differences and correlations to determine whether the spatial land use/land cover layout is balanced. The supplier valuation method was used to improve Xie’s [54] ESVs coefficient and to evaluate the four ESVs, after which a sensitivity analysis was conducted. Using the cold/hot spots analysis method in the ArcGIS spatial statistical tool (ESRI, Redlands CA, USA, available online: http://www.esri.com), a cold/hot spots spatial distribution map for the four ESVs was generated. From a regional perspective, changes in the ESVs in 26 cities in the Yangtze River Delta were analyzed, after which they were clustered into high and low ESVs regions to clearly show the regional imbalances in the spatial land use/land cover patterns. The research results provide decision support for the government to balance urban development and ecological environmental protection, and provide guidance for the development of ecological environmental spatial management and protection plans for the Yangtze River Delta.

## 2. Materials and Methods

### 2.1. Study Area and Data Source

**Figure 1:**
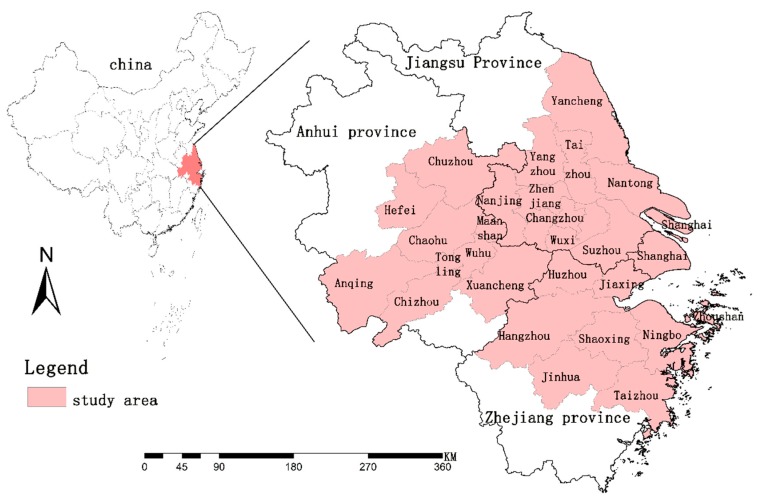
Study area.

The Yangtze River Delta urban agglomeration (Figure 1), located in the Yangtze River Economic Belt, is part of China’s latest stage of “reform and opening up”: The transformation and implementation of the new regional open development strategy. The area belongs to the alluvial plain and has a long history of agriculture. It is one of the most developed and densely populated industrial areas in China [55]. According to the “Changjiang Delta Urban Agglomeration Development Plan” approved by the State Council, the administrative scope of the Yangtze River Delta urban agglomeration includes Shanghai, Jiangsu Province (Nanjing City, Wuxi City, Changzhou City, Suzhou City, Nantong City, Yancheng City, Yangzhou City, Zhenjiang City, Taizhou City), Zhejiang Province (Hangzhou City, Ningbo City, Jiaxing City, Huzhou City, Shaoxing City, Jinhua City, Zhoushan City, Taizhou City), and Anhui Province (Hefei City, Wuhu City, Chaohu City, Maanshan City, Tongling City, Anqing City, Zhangzhou City, Chizhou City, Xuancheng City). It covers an area of 210,000 km^2^ with a population of 139.2 million and an average population density of 581 people per square kilometer. Shanghai is the core city of the Yangtze River Delta urban agglomeration.

**Figure 2:**
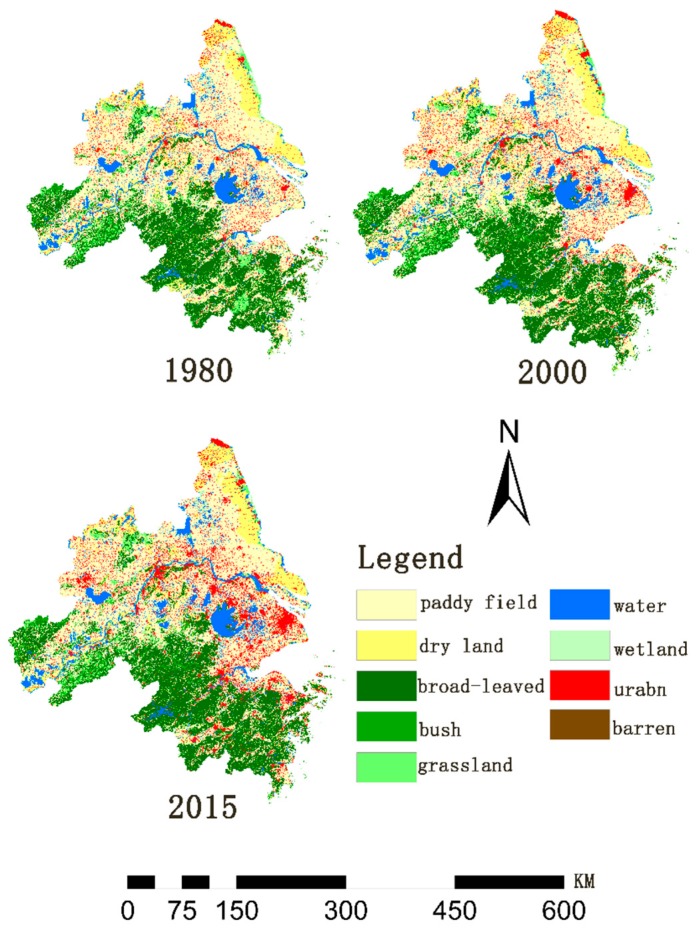
Spatial distribution of land use and land cover from 1980 to 2015.

The statistics on grain yield and price index used in this study are derived from the China Statistical Yearbook. The grain price data are from the government website of the National Development and Reform Commission. The land use data (Figure 2) are from the Resource and Environmental Science Data Center of the Chinese Academy of Sciences [56].

### 2.2. The Assessment Method of ESVs 

To date, the methods used to evaluate the ESVs in studies within the academic community have not been consistent [15]. The broader ESVs valuation method of “supplier valuation” was applied in the present study. The analysis presented in this paper draws on Xie’s [54] supplier valuation method and uses the product of the standard equivalence factor and the equivalent coefficient to determine the ESVs coefficient. The standard equivalence factor, as a benchmark for other ecosystem services, refers to the value provided by a natural ecological component of an ecosystem per unit of land area [54]. The standard equivalence factor is defined as the economic value of the grain yield of 1 hectare of farmland, which is obtained by multiplying the national grain yield and the national food price average during the study period. Adopting the classification of ecosystem and biodiversity economics [57], the ecosystem services are divided into four categories: Provisioning services, regulating services, habitat services, and cultural and amenity services [38]. Considering the representativeness of ecosystem services and its particular importance to the research field, according to present data, the existing value coefficient of different ecological land types in China further categorizes ecosystem services into food supply, water supply, material supply, climate regulation, air quality regulation, wastewater treatment, regulation of water flow, erosion protection, soil fertility maintenance, biodiversity, and aesthetic landscape. Eleven aspects were assessed for the ESVs generated in the Yangtze River Delta urban agglomeration. Since the research area of this study is an urban agglomeration, the research period extends from 1980 to 2015, so the timespan is long. Therefore, national-level statistical data were used for calculations, and the grain price used in the analysis is the average of the values during the study period. Taking into account the differences in food prices, a normalized food price index was used to correct the standard equivalence factor (Table 1):

(1) $$S_{c}=N_{PI}\times Y\times P$$

(2) $$C_{ij}=S_{c}\times E_{ij}$$

(3) $$\mathrm{ESV}=\overset{n}{\underset{i=1}{\sum }}\overset{m}{\underset{j=1}{\sum }}C_{ij}\times A_{i}$$

where $S_{c}$ is the standard equivalence factor; $N_{PI}$ is the normalized price index; *Y* is the average grain yield per unit (kg/ha) at the national level for the research period; *P* is the national average grain price per unit in CNY/kg; $C_{ij}$ is the value coefficient of the *i*-th land use type’s *j*-th ecosystem service; $E_{ij}$ is the equivalent coefficient of the i-th land use type’s *j*-th ecosystem services; $A_{i}$ is the area of the *i*-th land use type in hectares.

According to the Classification Standards for Land Use Types, land use types are divided into seven first-class land types, and there are six types of ESVs per unit area. According to the available land use data, farmland and forests are divided into subcategories: Paddy field and dry land for farmland; bush and non-bush for forests.

In this study, we introduced a simplified version of Aschonitis et al.’s method to establish the importance of each land use type based on its contribution to the total ESVs [58]; the importance values were then ranked:

(4) $$CS_{i}=\frac{C_{bi}\times A_{i}}{{\mathrm{ESV}}_{b}}$$

where $CS_{i}$ is the sensitivity factor of the *i*-th land use type; ${\mathrm{ESV}}_{b}$ is the total ESVs; $C_{bi}$ is the value coefficient of the *i*-th land type; $A_{i}$ is the area of the *i*-th land use type in hectares.

### 2.3. Global Spatial Autocorrelation

Global Moran’s *I* measures spatial similarity and was used to describe the ESVs’ distribution pattern of different units in the study area, i.e., spatial autocorrelation [59]. The spatial relevance of the ESVs of the Yangtze River Delta urban agglomeration can be measured by the Global Moran’s *I* Index [60]. The calculation formula for Global Moran’s *I* is as follows:

(5) $$I_{t}=\frac{n\sum_{i}\sum_{j}w_{i,j}z_{i}z_{j}}{S\sum_{i}z_{i}^{2}}$$

where $z_{i}$ and $z_{j}$ are the deviations of the *k*-th ecosystem service value (from the space unit *i*, *j*, respectively) from its mean, where *k* = 1, 2, 3, 4; $w_{i,j}$ is the spatial weight between spatial units *i* and *j*; *S* is the sum of all the elements of the weight matrix; and *n* is the total number of spatial units. The range for Global Moran’s *I* index is [−1, 1]. The exponent with this variable greater than 0 presents an overall positive spatial correlation; if it is less than 0, it means there is a negative correlation; if it is equal to 0, it means that there is no correlation. The importance of each calculated Global Moran’s *I* index can be tested by *Z* statistics [60].

### 2.4. Cold/Hot Spots Analysis

In order to detect the high and low values and the degree of clustering of potential ESVs in different regions, the Getis–Ord $G_{i}^{*}$ statistic was calculated for different ESVs using the spatial statistical tool in ArcGIS, the cold/hot spots analysis tool. The higher the Z-score of the $G_{i}^{*}$ index, the closer the aggregation of high values (hot spots) for a given ecosystem service [61] and the higher the values of the attributes around the unit. The lower the score, the tighter the aggregation of low values (cold spots), the lower the value of the attributes around the unit, and the lower the ecosystem services supply value [62]. The $G_{i}^{*}$ index calculation formula is as follows:

(6) $$G_{i}^{*}=\frac{\sum_{j=1}^{n}w_{i,j}x_{j}-\bar{X}\sum_{j=1}^{n}w_{i,j}}{S\sqrt{\frac{\left[n\sum_{j=1}^{n}w_{i,j}^{2}-{\left(\sum_{j=1}^{n}w_{i,j}\right)}^{2}\right]}{n-1}}}$$

where $x_{j}$ is the value of the *k*-th ecosystem service of space unit *j*; $w_{i,j}$ is the spatial weight between spatial units *i* and *j*; *n* is the total number of spatial units.

## 3. Results and Analysis

### 3.1. Analysis of the Changes in the ESVs Resulting from LUCC

**Figure 3:**
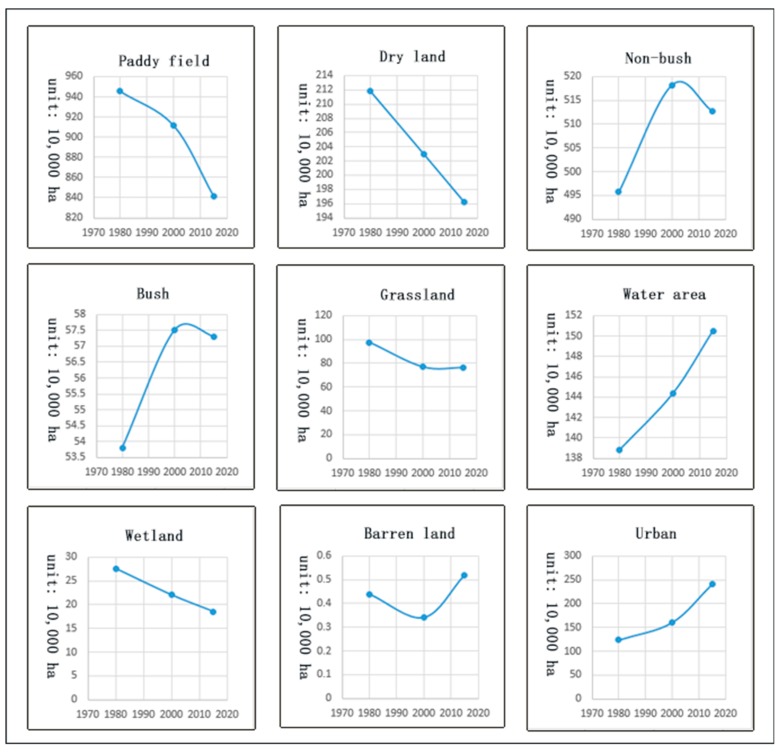
Time trends of different land use/cover types.

During the study period, land use/land cover underwent major changes (Figure 3). From 1980 to 2015, farmland (paddy field and dry land), grassland, and wetland were gradually decreasing; the trend of decreasing paddy field area gradually increased and the trend of decreasing grassland area gradually weakened. The area of water and towns gradually increased, and both bush and non-bush forests first increased and then decreased. From 1980 to 2000, the area of bush and non-bush forests increased greatly, while wasteland first decreased and then increased.

**Figure 4:**
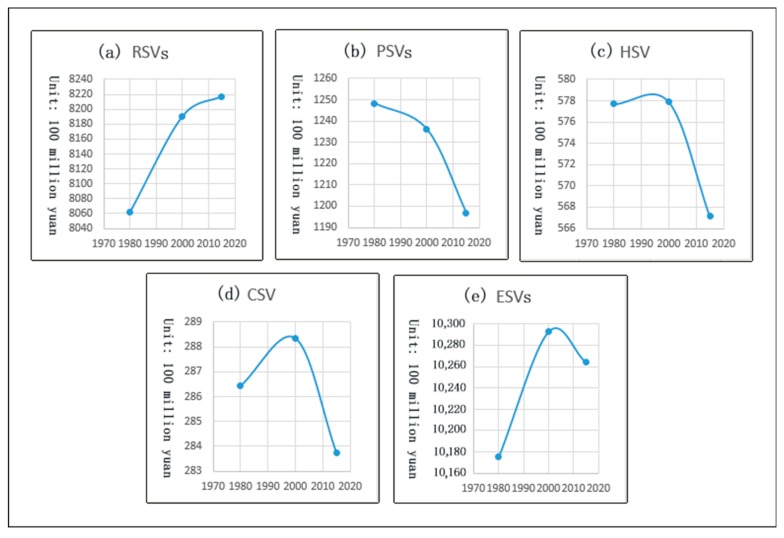
Time-varying trends of ESVs in the Yangtze River Delta urban agglomeration. (**a**) Regulating services values (RSVs); (**b**) provisioning services values (PSVs); (**c**) habitat services value (HSV); (**d**) cultural and amenity services value (CSV); and (**e**) total ecosystem services values (ESVs).

Figure 4 shows the ESVs trends in the Yangtze River Delta urban agglomeration at three time points in the period between 1980 and 2015. The PSVs (provisioning services values) were gradually decreasing. It can be seen from the interval between the two time points that the ESVs decreases most during the period from 2000 to 2015. The RSVs (regulating services values) were gradually increasing, the HSV (habitat services value) and the CSV (cultural and amenity services value) first increased and then decreased. Habitat services gradually started decreasing after 2000, which is an indirect indication that the quality of habitats is declining. The total ESVs first increased and then decreased. Before 2000, the ESVs increased significantly, and then it decreased. This is an early warning sign of the destruction of ecological land, and it needs attention.

Comparing Figure 3 and Figure 4, the temporal change trend of forest area (bush and non-bush) is similar to the change trend of the total ESVs, indicating that the change in forest area is the main factor affecting the change in the total ESVs [63]. The PSVs in the decreasing farmland (paddy field and dry land) also decreased, indicating that changes in farmland have a great impact on provisioning services. Regulating services, habitat services, and cultural and amenity services may be affected by a combination of these land use/cover types.

During the period from 1980 to 2000, the farmland (paddy field and dry land) and grassland areas decreased the most: The farmland area decreased by 456,600 hectares, and the ecological service value provided by farmland decreased by about 7.437 billion yuan. Although the wetland only decreased by 55,300 hectares, the value of the ecological services it provides decreased by about 7250.9 million yuan. Although the water area only increased by 55,300 hectares, the value of the ecological services it provides increased by 175.083 billion yuan. The disproportionately large effects of the changes in the wetland and water area are because these land types are important ecological protection barriers and are associated with higher value factors. The area of forests (bush forests and non-bush forests) increased by 261,600 hectares, and the value of its ecological services increased by 14.481 billion yuan. The urban area increased by 366,000 hectares. The total ESVs increased by 11.765 billion yuan, which was mainly due to the increase in forest and water area. From 2000 to 2015, the farmland area decreased by 766,600 hectares, and the ESVs decreased by 14.251 billion yuan, about double that of the previous period. The water area increased by 61,400 hectares, and the urban area increased by 801,400 hectares. During the study period, except for a small increase in the area of wasteland, the area of the remaining land use/cover types was reduced. The value coefficient and the change in the area of the wasteland were small, and the ESVs provided was low. So, the total ESVs decreased by 2.818 billion yuan. During the period of 1980–2015, a large amount of farmland was transformed into towns, causing serious degradation of farmland and severely affecting provisioning services (Table 2).

**Figure 5:**
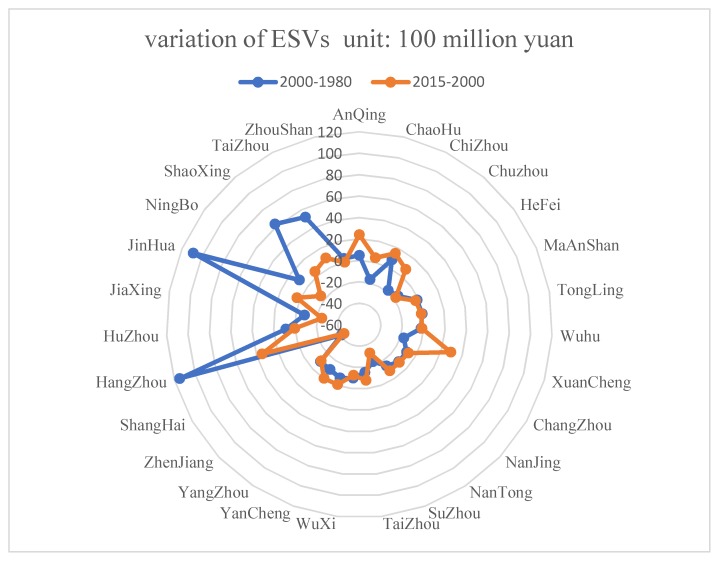
Changes in ESVs in different urban areas of the Yangtze River Delta urban agglomeration.

According to the radar chart, the regional variation in the ESVs is significant (Figure 5). From 1980 to 2000, the largest values’ variation of ecosystem services was in Jinhua City, Hangzhou City, Ningbo City, and Taizhou City, and it was also positive. The ESVs of Huzhou City, Chizhou City, Anqing City, and Zhoushan City increased slightly, and other cities experienced a decrease. From 2000 to 2015, the values’ variation in ecosystem services was the largest in Hangzhou, Xuancheng, and Anqing. The values’ variation of ecosystem services was positive due to a small increase in ESVs in Taizhou, Zhoushan, Shaoxing, Jinhua, Huzhou, Zhangzhou, Chizhou, and Chaohu, while the ESVs in the other cities decreased. There were 14 cities with reduced ecosystem services during the two study periods: Shanghai, Zhenjiang, Yangzhou, Yancheng, Wuxi, Taizhou, Suzhou, Nanjing, Nantong, Changzhou, Wuhu, Tongling, Maanshan, and Hefei. Among them, Shanghai had the largest reduction, and its ESVs continue to decrease.

### 3.2. Spatial Autocorrelation Analysis and Correlation Analysis of Four Ecosystem Services

It can be seen from Table 3 that the Moran I index of the ESVs in the study area is greater than 0, and the Z-score is higher than the standard 1% significance level value of 1.65. This indicates that the spatial distribution of ESVs in the study area has a strong spatial autocorrelation—it is aggregated in space. The PSVs index I and the Z-score showed a downward trend. The RSVs index I and the Z-score showed an upward trend, and the CSV index I and the Z-score showed an upward trend.

The Pearson correlation coefficients (5% significance level) for the four potential ecosystem services are presented in Table 4. Provisioning services were negatively correlated with habitat services and cultural and amenity services. Regulating services were weakly positively correlated with provisioning services and were significantly positively correlated with habitat services and cultural and amenity services. The correlation coefficients were 0.219, 0.857, and 0.871, respectively. Habitat services were significantly positively correlated with cultural and amenity services; these two services had the highest correlation (0.998).

### 3.3. Evolution of the Spatial Pattern Of ESVs Based on Cold/Hot Spots 

**Figure 6:**
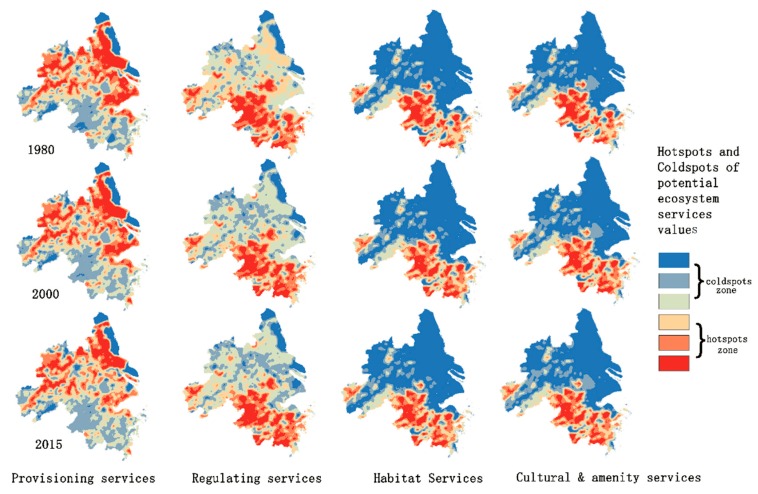
Potential ecosystem services supply value based on the $G_{i}^{*}$ index Z score.

The Getis–Ord $G_{i}^{*}$ statistic [64] is one of the Local Indicators of Spatial Association (LISA) measures [65]. Calculating Getis–Ord $G_{i}^{*}$ statistics can identify hot spots and cold spots of the potential supply of each ecosystem service. Cold/hot spots are areas with spatial clustering features and high/low ecosystem service supply values [62]. The cold/hot spots analysis results enable spatial and visual comparisons among ecosystem services [66]. The cold/hot spots analysis tool of ArcGIS was used to calculate the spatial distribution of the high and low clusters of ESVs of the four different ecological service types (Figure 6). Higher Z-scores indicate a higher intensity of ESVs clustering. Lower Z-scores indicate a lower intensity of ESVs clustering.

From the overall spatial layout, the hot spots of provisioning services mainly aggregated in the northern part of the Yangtze River Delta urban agglomeration, and the cold spots accumulated in the southern part of the Yangtze River Delta urban agglomeration and along the coast. In the time dimension, from 1980 to 2015, provisioning services hot spots showed a trend of shrinking and aggregating, and the high-ESVs area was decreasing. The hot spots of regulating services were mainly concentrated in the southern part of the Yangtze River Delta urban agglomeration. There were a few hot spots in the western and central regions. The cold spots were mainly concentrated in the north and east of the Yangtze River Delta urban agglomeration along the coastal area. From 1980 to 2015, the hot spots of regulating services showed a trend of shrinking and aggregating, the cold spots area was spreading, and the low-ESVs area was increasing. The value of habitat services was divided into a low-ESVs north and a high-ESVs north. There are almost very large cold spots in the north. Cultural and recreational services have distributions similar to that of habitat services. From 1980 to 2015, the hot spot area of habitat services and cultural and amenity services gradually decreased, and the cold spots area gradually expanded.

From the analysis of the local spatial layout, provisioning services hot spots gathered in the administrative areas of Anhui Province, Jiangsu Province, and Shanghai, indicating that they contributed a high PSVs. Where the farmland was concentrated in these areas, the forest area was small. There were also large cold spots in Jiangsu Province, mainly in coastal cities. The area of service value in these cities decreased, and the trend of cold spots spread in Shanghai was the most obvious. According to the land use/cover change thematic map (Figure 2), Shanghai’s urban expansion has been the most obvious over time. In the past 35 years, the urban area has been encroaching on and occupying the farmland in Shanghai, and farmland degradation is the most serious in this location. Several concentrated cold spots areas in Zhejiang Province have gradually disappeared. The hot spots of regulating services were mainly concentrated in Zhejiang Province, while the proportion of forest coverage in Zhejiang Province was relatively high. The large cold spots area in Jinhua City disappeared and was replaced by hot spots. The hot spots of habitat services and cultural and amenity services were mainly concentrated in Zhejiang Province, and a small number were gathered in Anhui Province.

### 3.4. Analysis of the Importance of Land Use Types on the ESVs

To reflect the contribution from each land use type to the ESVs, the importance values of different land use types based on their contribution to the total ESVs were calculated. The results are listed in Table 5, and their importance values are ranked. In the study area, the water area is the most important for the total ESVs, followed by non-shrub forests, with paddy fields ranked third. Drylands, shrubs, grasslands, and wetlands are less important, and the importance of barren land is almost zero. This is because the value coefficient of the water area is much higher than that of the other land types, while the area of the wasteland is smaller. For the time points, 1980, 2000, and 2015, the sensitivity coefficient of the water area increased, the sensitivity coefficient of the paddy field decreased, and the sensitivity coefficient of the non-shrub forest first increased and then decreased. These changes in the sensitivity coefficients reflect the changes in area: The water area increased, the paddy field area decreased, and the non-shrub forest area first increased and then decreased.

In the Yangtze River Delta urban agglomeration, water, non-shrub forest, and paddy fields are important indicators when considering the total ESVs. In general, when planning land use in the region, incorporating the importance of different land use types based on their contribution to the total ESVs is necessary.

## 4. Discussion

### 4.1. Data Method Restrictions

The Yangtze River Delta urban agglomeration is located on the East coast of China. Because of the difficulties in acquiring all the needed data, the research results did not include the ocean ESVs. Further, to better unify the study area, the impact of sea reclamation on ecological services was also not included; however, these will be included in future research. The research method was based on Constance and Xie’s supplier valuation method, for which the value coefficient was improved to increase the accuracy of the results. This method has been widely used to assess the monetary costs of ecosystem services, and has proven to be a more effective method than assessing the benefits [67]. It has also been found that more extensive and more accurate field survey data is needed, if scholars do more accurately assess each type of ESVs in terms of ecosystem. This survey data not only eliminates some of the biases inherent in the overgeneralization and simplification of LULC [68], but also reduces the uncertainty in the analysis [69].

### 4.2. Causes of ESVs Changes in the Study Area

In the study area, changes in the ESVs were caused by changes in the type of land use. For example, in order to improve the ecological environment, the Zhejiang Provincial Government proposed in 1989 a strategic plan of “two years of preparation, five years to eliminate the barren hills, and ten years to green Zhejiang” [70]. This is the largest natural transformation activity in the history of Zhejiang Province. In 2000, the Zhejiang Provincial People’s Government announced that the strategic goal of “Greening Zhejiang for Ten Years” was achieved on schedule. Afforestation amounted to about 582,000 hectares, which is equivalent to more than 1/10 of the province’s existing forest area. All the barren hills suitable for planting forests in the province have been eliminated. The forest land area, forest stock volume, and forest coverage rate of the province all met the plan’s targets. In the same year, the Anhui Provincial Government proposed the “five eight” greening planning target of “Five-year elimination of barren hills and greening of Anhui in eight years” [71]. During the “five eight” afforestation period, a total of 324,000 ha of economic forestry was completed, accounting for 39.8% of the area of artificial afforestation, an increase of more than 20% above the quantity before the implementation. The following year, the State Council promulgated the “Outline of National Afforestation and Greening Plan from 1989 to 2000” to promote greening and afforestation throughout the country. This led to a sharp increase in the area of forestland within the study area before 2000. From 1980 to 2000, the state adjusted the industrial structure of the Yangtze River Delta region, especially by increasing the aquaculture area, which led to an increase in the water area. Before 2000, the increase of the forest and water area was the main cause of the total ESVs growth. After 2000, a series of national economic and social development, as well as urban and rural construction policies, such as the “Ninth Five-Year Plan”, resulted in the Yangtze River Delta and the Yangtze River Delta urban agglomeration ranking among the top seven economic zones, leading to faster urban residential construction and a rapidly increasing construction land. As the process of economic development and urbanization accelerates, a growing number of people are moving to cities, causing the towns to expand. The enhancement of urbanization has taken up a large amount of farmland, forest, and grassland in the Yangtze River Delta urban agglomeration, which has reduced the total ESVs.

The method used in this study, cold/hot spots analysis, can well represent the spatial difference in ecosystem services, and many scholars apply it to the spatial analysis of ecosystem services [72,73,74]. In the study area, the spatial differences between provisioning services and regulating services may have been caused by the uneven distribution of land use/land cover. The hot spots of provisioning services were in the northern part of the Yangtze River Delta because the provisioning services were mainly provided by farmland as a crop production area [75], and farmland is distributed in the north of the Yangtze River Delta urban agglomeration (Figure 1) and covers Jiangsu Province, Anhui Province, and Shanghai. The regulating services were mainly provided by forests, which are distributed in the southern part of the Yangtze River Delta urban agglomeration, mainly in Zhejiang Province. The habitat services and cultural and amenity services were found to have similar spatial distributions, which reflected the synergy between their ecological services [76]. Some scholars have found that in the middle and lower reaches of the Yangtze River, there are hot spots of species [77], and more species may live in the middle and lower reaches of the Yangtze River. The forest resources in the middle and lower reaches of the Yangtze River are abundant, indicating that the habitat quality is good, which is likely because good quality habitats are beneficial for the tourist culture. Nowadays, urbanization is becoming ever more serious. In some areas, the habitat area is reduced, biodiversity is threatened, land use changes are leading to a fragmented habitat distribution, and species are threatened with extinction [77,78,79]. In response to these environmental threats, scholars have developed appropriate decision support systems to incorporate biodiversity, ecosystem services, and land use change [19].

Some scholars have found that reductions in farmland and forest areas can lead to a decline in the ESVs. As urban land tends to be taken from high-quality farmland, forests, and grasslands, these land use changes result in ESVs losses. Additionally, it was found that the increase in forest and water areas from 1980 to 2000 resulted in an increase in the ESVs. Therefore, these changes in the ESVs were strongly related to changes in the farmland, forests, and water bodies. Further, the results of this study are consistent with those of Xin and Meng et al. [48,80]. Correlation analyses have been commonly used to explore ecosystem services trade-offs and synergies [81,82]; for example, Roces-Díaz used correlation analysis to determine the synergy between cultural and amenity services and regulating services. Therefore, the findings validated past research as they also indicated that there was a synergy between the cultural and amenity services, the regulating services, and the habitat services.

## 5. Conclusions and Policy Recommendations

LUCC maps were used to analyze the potential supply of four ecosystem services and evaluate for four ESVs in the Yangtze River Delta urban agglomeration. The cold/hot spots analysis tool in ArcGIS was used to calculate the spatial distributions for the high and low ESVs clusters for the four ecosystem services types. The spatial difference in four ESVs brought about by LUCC under rapid urbanization was analyzed. In most areas, and especially in the northern part of the Yangtze River Delta, there has been a significant increase in urban land use, which has led to a decline in high-quality farmland, forests, and grasslands, all of which have resulted in ESVs losses. The ESVs changes had obvious regional differences. The ESVs in Hangzhou and Jinhua City had noticeable increases; however, Shanghai had the largest ESVs declines. The spatial distributions for the four ESVs differed significantly. The provisioning services hot spots were primarily in the northern part of the Yangtze River Delta urban agglomeration, and the cold spots were primarily in the southern part. The regulating services hot spots were mainly concentrated in the southern part, there were a few hot spots in the western and central regions, and the cold spots were in the north and east along the coast. The habitat services value also show that the north is low and the south is high. The cultural and amenity services had a similar distribution to the habitat services. However, the analysis showed that over time, the hot spots were gradually shrinking and the cold spots were increasing. The Pearson’s correlation analysis showed the interrelationships between the four ecosystem services, and reflected their trade-offs and synergies. The provisioning services were negatively correlated with the habitat services and the cultural and amenity services. There was a weak positive correlation between the regulating services and the provisioning services, and a significant positive correlation with habitat services and the cultural and amenity services. Finally, the habitat services were significantly positively correlated with the cultural and amenity services. In the Yangtze River Delta urban agglomeration, the water area was the most important for the total ESVs, followed by non-bush forest. Paddy field was ranked third. Dryland, bush, grassland, and wetland were less important. The importance of barren land was almost zero. Therefore, water, non-bush forest, and paddy fields are important indicators when considering the total ESVs.

In recent years, the government has built a shelterbelt system on the banks of the Yangtze River, the Huaihe River, the Beijing-Hangzhou Grand Canal, and other lakes and has established a soil erosion control project in the small watershed area, which has improved the RSVs. Additionally, the Chinese government has formulated a policy of returning farmland to forests and returning farmland to grassland. These policies have helped ecological restoration. However, due to rapid urbanization, the current ecological environment is still deteriorating. It is still important to balance the protection of cultivated land and forest protection during the process of regional land development and utilization. The ESVs in the Yangtze River Delta urban agglomeration are declining and shows differences in space, which also reflects the disproportionate spatial pattern of land use structure. When formulating regional land use planning policies, ecological protection should be given top priority, and the ESVs should be incorporated into decision making. The importance of the contribution of different land use types to the total ESVs in the region should be considered. Therefore, within the study area, the following two suggestions are proposed based on the research results: (1) According to the research results, the contribution of the water area to the total ESVs is the most important. However, in recent years, due to rapid urbanization, agricultural pollution has increased due to, for example, the use of chemical fertilizers and pesticides and the expansion of livestock and poultry farming. An excessive amount of industrial sewage discharge exacerbates the seriousness of water pollution. Although the government has formulated a “transboundary water body joint guarantee operation” as a response to the insufficiencies in environmental protection laws, the punishment for illegal sewage disposal is not severe enough, so the challenge of protecting water from pollution still exists. Therefore, for the illegal discharge of pollutants, a high penalty system would be conducive to the protection of water. (2) The expansion of the urban scape has occupied a large amount of farmland, which has reduced the ESVs contributed by farmland. Shanghai, the largest urban area in the Yangtze River Delta, had an average annual expansion of about 3225 hectares from 1980 to 2000; however, from 2000 to 2015, its annual expansion was about 5907 hectares, and the urban annual expansion area increased by 83.16%. Therefore, it is very necessary to strictly control the construction scale of megacities and large cities by designating areas of permanent farmland as boundaries to urban development. (3) In terms of space, the western and southern parts of Zhejiang Province are rich in forest resources, and hot spots for regulating services, habitat services, and cultural and amenity services are concentrated in these areas. It is necessary to list these areas as ecological protection barriers and to define ecological corridor protection areas in the Yangtze River Basin to protect the water resources along the Yangtze River basin.

## Figures and Tables

**Table 1:** The ecosystem services values (ESVs) per unit area of terrestrial ecosystems, yuan/ha.

		Farmland		Forest		Grassland	Water Area	Wetland	Barren Land
		Paddy Field	Dry Land	Bush	Non-Bush	Grassland	Water Area	Wetland	Barren Land
Provisioning services	Food	3427.934	2142.459	478.9026	781.3674	554.5188	2016.432	1285.475	0
	Material	226.8486	1008.216	1083.832	1789.583	831.7782	579.7242	1260.27	0
	Water	2974.237	50.4108	554.5188	932.5998	453.6972	20,895.28	6528.199	0
Regulating services	Air quality regulation	2797.799	1688.7618	3553.961	5923.269	2873.416	1940.816	4789.026	50.4108
	Climate regulation	1436.708	907.3944	10,661.88	17,719.4	7612.031	5772.037	9073.944	0
	Wastewater treatment	428.4918	252.054	3226.291	5015.875	2520.54	13,989	9073.944	252.054
	Regulation of water flow	6855.869	680.5458	8443.809	8847.095	5570.393	257,700	61,072.68	75.6162
	Erosion protection	25.2054	2596.1562	4335.329	7208.744	3503.551	2344.102	5822.447	50.4108
	Soil fertility maintenance	478.9026	302.4648	327.6702	554.5188	277.2594	176.4378	453.6972	0
Habitat services	Biodiversity	529.3134	327.6702	3957.248	6553.404	3201.086	6427.377	19,836.65	50.4108
Cultural and amenity services	Aesthetic landscape	226.8486	151.2324	1739.173	2873.416	1411.502	4763.821	11,922.15	25.2054

**Table 2:** Changes in land use/cover area and ESVs in the Yangtze River Delta urban agglomeration from 1980 to 2015.

Land Use/Cover Type	1980–2000 Change				2000–2015 Change			
	Area (10,000 ha)	%	Value (100 Million Yuan)	%	Area (10,000 ha)	%	Value (100 Million Yuan)	%
Paddy field	−33.71	−3.565	−65.425	−3.565	−69.96	−7.673	−135.779	−7.673
Dry land	−8.85	−4.178	−8.945	−4.178	−6.7	−3.301	−6.772	−3.301
Bush	3.73	6.934	14.309	6.934	−0.22	−0.382	−0.844	−0.382
Non Bush	22.43	4.524	130.505	4.523	−5.37	−1.036	−31.260	−1.036
Grassland	−20.1	−20.643	−55.359	−19.915	−0.71	−0.919	−2.045	−0.918
Water	5.53	3.984	175.083	3.983	6.14	4.254	194.396	4.253
Wetland	−5.53	−20.044	−72.509	−20.043	−3.5	−15.866	−45.892	−15.865
Barren land	−0.1	−22.727	−0.005	−22.727	0.18	52.941	0.009	52.941
Urban	36.6	29.50899	0	0	80.14	49.891	0	
Total value change			117.654	1.156	Total value change		−28.188	−0.274
total area			2095.06		total area		2095.06	

**Table 3:** ESVs in the Yangtze River Delta urban agglomeration Moran I spatial autocorrelation index.

	Spatial Autocorrelation of ESVs							
	Provisioning Services		Regulating Services		Habitat Services		Cultural and Amenity Services	
	Index I	Z Score	Index I	Z Score	Index I	Z Score	Index I	Z Score
1980	0.325859	15.76806351	0.341315	13.54484010	0.443899	21.47996642	0.433891	21.3177653
2000	0.296663	14.35484476	0.344935	16.69057657	0.471953	22.83665061	0.456325	21.7954661
2015	0.264615	12.80752928	0.345942	16.743576	0.478471	22.97422287	0.474623	22.9187627

**Table 4:** Correlation coefficients of four potential ecosystem services.

Ecosystem Services	Regulating Services	Provisioning Services	Habitat Services	Cultural and Amenity Services
Regulating services	1	-	-	-
Provisioning services	0.219	1	-	-
Habitat services	0.857	−0.135	1	-
Cultural and amenity services	0.871	−0.150	0.998	1

**Table 5:** Sensitivity coefficient of ESVs in the Yangtze River Delta urban agglomeration.

	1980		2000		2015	
	Sensitivity Coefficient	Rank	Sensitivity Coefficient	Rank	Sensitivity Coefficient	Rank
Paddy field	0.1785	3	0.1721	3	0.1589	3
Dry land	0.0208	6	0.0199	7	0.0193	7
Bush	0.0201	7	0.0215	6	0.0214	6
Non-bush	0.2801	2	0.2927	2	0.2897	2
Grassland	0.0273	5	0.0217	5	0.0215	5
Water area	0.4275	1	0.4445	1	0.4634	1
Barren land	0	8	0	8	0	8
Wetland	0.0352	4	0.0281	4	0.0237	4
